# A Novel Truncating Mutation in PAX1 Gene Causes Otofaciocervical Syndrome Without Immunodeficiency

**DOI:** 10.1007/s12031-023-02170-7

**Published:** 2023-11-04

**Authors:** Nagham M. Elbagoury, Asmaa F. Abdel-Aleem, Wessam E. Sharaf-Eldin, Engy A. Ashaat, Mona L. Esswai

**Affiliations:** 1 Medical Molecular Genetics Department, Human Genetics and Genome Research Institute, National Research Centre, Cairo, Egypt; 2 Clinical Genetics Department, Human Genetics and Genome Research Institute, National Research Centre, Cairo, Egypt

**Keywords:** Otofaciocervical syndrome, Intellectual disability, Facial dysmorphism, Whole exome sequencing, *PAX1* gene

## Abstract

Otofaciocervical syndrome (OTFCS) is a rare genetic disorder of both autosomal recessive and autosomal dominant patterns of inheritance. It is caused by biallelic or monoallelic mutations in *PAX1* or *EYA1* genes, respectively. Here, we report an OTFCS2 female patient of 1st consanguineous healthy parents. She manifested facial dysmorphism, hearing loss, intellectual disability (ID), and delayed language development (DLD) as the main clinical phenotype. The novel homozygous variant c.1212dup (p.Gly405Argfs*51) in the *PAX1* gene was identified by whole exome sequencing (WES), and family segregation confirmed the heterozygous status of the mutation in the parents using the Sanger sequencing. The study recorded a novel *PAX1* variant representing the sixth report of OTFCS2 worldwide and the first Egyptian study expanding the geographic area where the disorder was confined.

## Introduction

Otofaciocervical syndrome (OTFCS) is a rare group disorder that is subclassified into two types: autosomal recessive OTFCS2 (MIM: 615,560) and autosomal dominant OTFCS1 (MIM: 166,780) due to gene mutations in *PAX1* and *EYA1*, respectively (Patil et al. 2018). OTFCS is characterized by a combination of facial dysmorphisms, shoulder girdle anomalies, ear abnormalities accompanied by hearing loss, and intellectual disability (Pohl et al. 2013). It showed some overlapping clinical features with branchiootorenal spectrum disorders (BSDs), including branchiootic syndrome (BOS, MIM#60,258) and branchiootorenal syndrome (BORS, MIM#113,650) (Gana et al. 2019).

OTFCS2 is caused by a biallelic *PAX1* mutation. The hypofunctional homozygous missense mutation c.497G < T (p.G166V) was described in the first OTFCS2 family who presented with facial dysmorphism, nasolacrimal duct and shoulder girdle abnormalities, vertebral anomalies, ear malformations, hearing loss, and mild intellectual disability (Pohl et al. 2013). However, more severe expanded features, including thymus aplasia, T-cell immunodeficiency, and recurrent infections, were observed in other successive studies (Paganini et al. 2017; Yamazaki et al. 2020; Sherlaw-Sturrock et al. 2022). To date, only fourteen affected individuals with OTFCS2 from six different families have been documented. *PAX1* is a member of the paired-box (PAX) family that has already been classified into four main subfamilies, “Pax1 and Pax9; Pax2, Pax5, and Pax8; Pax3 and Pax7; Pax4 and Pax6,” according to their expression patterns, genomic organization, and the paired domain sequence. *PAX1* is localized at chromosome 20p11.22, including 5 exons and encoding a 534 amino acid protein (NP_006183.2). The most conserved functional motif of *PAX1* and its family is the 128 amino acid (98–226) paired-box domain (PD), which is responsible for DNA-binding activity (Blake and Ziman 2014; Thompson et al. 2021). *PAX1* encodes a transcription factor protein specifically expressed during the development of the skeleton, thymus, and parathyroid glands (Wu et al. 2022). Experimentally, mice with *PAX1* deficiency showed vertebral column anomalies and different degrees of thymic hypoplasia (Yamazaki et al. 2020).

Human *PAX1* mutations are also involved in the Klippel-Feil (KFS) syndrome and oculo-auriculo-vertebral syndrome (OAVS) (Carter et al. 2022). The hypermethylated *PAX1* promoter in esophageal squamous cell carcinoma suggests *PAX1* as a tumor suppressor gene, highlighting its multifaceted roles in the human body (Nishiyama and Nakanishi 2021). Here, we present the clinical and molecular findings of the first Egyptian patient with OTFCS2 representing the sixth reported case worldwide.

## Material and Methods

### Patient Recruitment and Ethical Approval

A 13-year-old female patient was referred to the Multiple Congenital Anomalies (MCAs) Clinic, Center of Excellence for Human Genetics at the National Research Centre (NRC). Written informed consent was obtained from the parents according to the Declaration of Helsinki. The study was approved by the Medical Research Ethics Committee of the NRC (ID: 19,261).

### Molecular Study

DNA was extracted from the peripheral blood lymphocytes using PAXgene Blood DNA (QIAGEN, Germany) according to the manufacturer’s protocol. Exome mapping was applied using the xGen Exome Research Panel v2 (Integrated DNA Technologies, Coralville, IA, USA), and sequencing was applied using the NovaSeq 6000 (Illumina, San Diego, CA, USA). Approximately 98.9% of the targeted bases were covered to a depth of ≥ 20x. All detected reads were mapped against the human reference genome (GRCh37/hg19) followed by validation of small indels and nucleotide variants using GATK best practices guidelines (Pollard et al. 2009). Variants were annotated using ANNOVAR for location and function prediction (Wang et al. 2010). Only variants deemed relevant to the patient’s clinical phenotypes were evaluated. The detected novel-related variant was examined in different databases, including ClinVar (Landrum et al. 2016), gnomAD (Karczewski and Francioli 2017), HGMD (Stenson et al. 2003), 1000 Genomes (http://browser.1000genomes.org/index.html), ExAC (http://exac.broadinstitute.org/), and GME Variome (Scott et al. 2016). The pathogenicity of the novel variant was examined by several functional prediction tools including mutation taster (Schwarz et al. 2014), CADD (Rentzsch et al. 2019), DDIG-in (Zhao et al. 2013), and TransPPMP (Nie et al. 2022). Additionally, PhyloP 100 and PhastCons 100 were checked for variant evolutionary conservation using multiple alignments based on 100 vertebrate genomes, including humans. Exon 4 of *PAX1* (GenBank: NM_006192.4) was amplified utilizing standard PCR cycling conditions with specifically designed primers (**F**: 5′-TAATGGATGGGCACAGGACG-3′ and **R**: 5′-AAGTGGAGGGGACAGTCTTG-3′). The Sanger sequencing was applied using an ABI 3500 Genetic Analyser (Applied Biosystems) for variant validation and cosegregation.

## Results

### Clinical Results

**Fig. 1:**
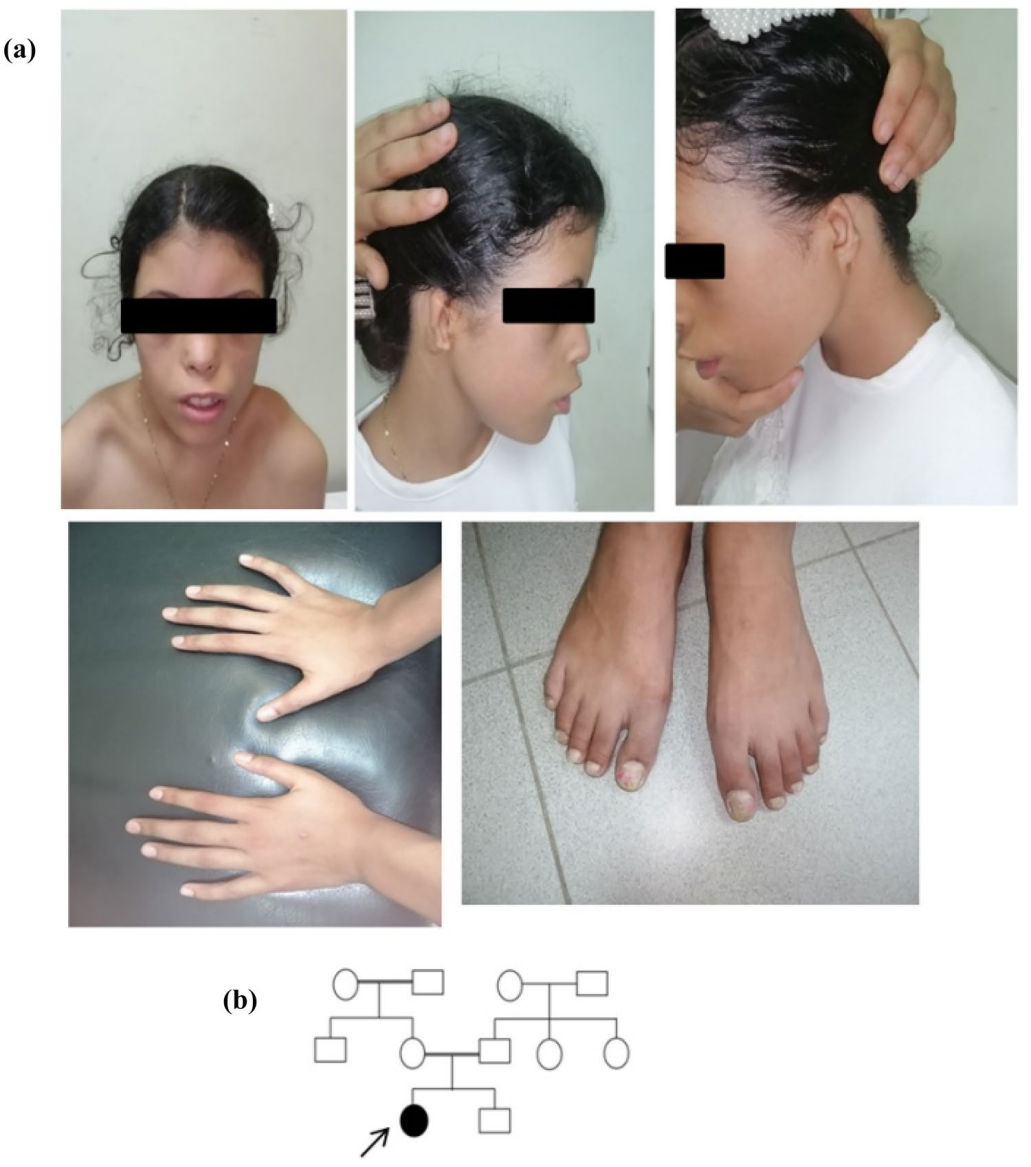
**a** Images of the proband demonstrate a long face, sparse outer third of the eyebrows, long lashes, hypertelorism, down slanting palpebral fissures, broad nose, bilateral microtia, bilateral low set ears, short neck, bilateral sloping shoulder, and toenail dystrophy. **b** Family pedigree: the black arrow denotes to the affected proband

The patient was the first offspring of a 1st-degree consanguineous parent who had another healthy sibling (Fig. 1). The pregnancy history was uneventful, and the proband had a low birth weight (2 kg), as mentioned by the parents. She had a history of normal motor developmental milestones with no history of recurrent infection. A general clinical examination revealed delayed mental milestones and speech development. The patient was dysmorphic with characteristic facial features, including a long face, sparse outer third of the eyebrows, long lashes, hypertelorism, down slanting palpebral fissures, broad nose, bilateral microtia, bilateral low set ears, short neck, bilateral sloping shoulder, and toenail dystrophy, but no abnormalities were detected in the hands. Anthropometric measurements were normal according to the patient’s age (weight = 36 kg, height = 141 cm, and head circumference = 53 cm). Neurological examination showed normal muscle tone and reflexes. The auditory brainstem response (ABR) test showed bilateral sensorineural hearing loss (SNHL). Echocardiography and pelvic-abdominal ultrasound were normal. Fundoscopic and genital examinations also revealed normal findings.

### Molecular Results

**Fig. 2:**
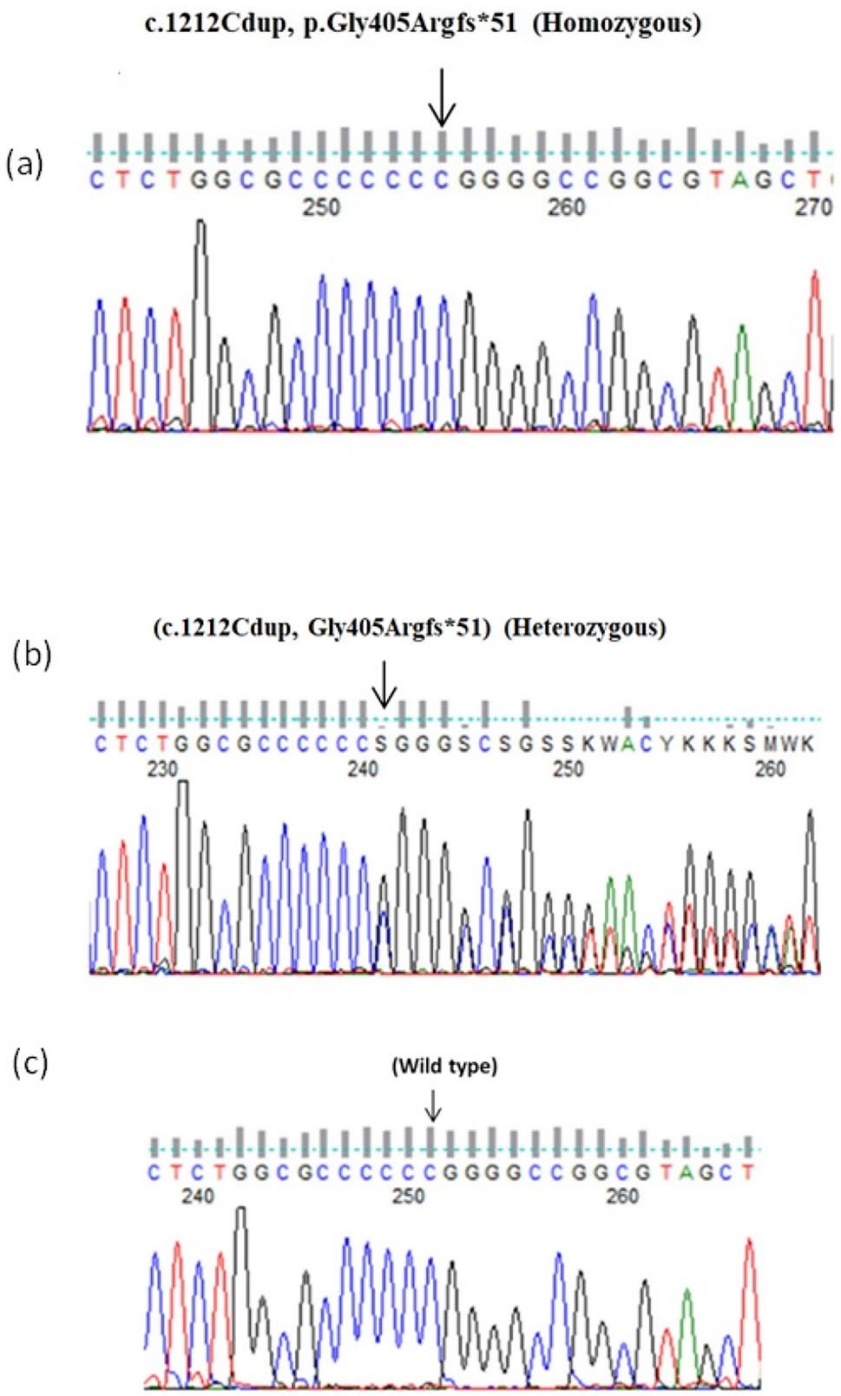
*PAX1* genotypes of the included subjects: **a** the proband homozygous mutation (c.1212Cdup, p.Gly405Argfs*51) in *PAX1* gene, **b** heterozygous status of the revealed mutation in parents, and **c** wild-type sequence

A homozygous variant was identified in *PAX1*, one base duplication of cytosine at amino acid position 405 (NM_006192.4:c.1212dup; p.Gly405Argfs*51) in exon 4, creating a premature stop codon and truncated protein at position 456 in exon 5 of the altered sequence. The Sanger sequencing confirmed the detected homozygous variant in the proband and the carrier status in the parents, while the healthy sibling showed wild-type alleles (Fig. 2). The variant has not been detected in gnomAD, 1000 Genomes, HGMD, ClinVar, or ExAC. It has been classified as pathogenic according to the American College of Medical Genetics (ACMG) recommendations for variant classification. The variant deleterious effect has been also supported by several in silico prediction tools with relatively high conservation scores (Phylop100 = 5.324 and PhastCons 100 = 1.000) (Table 1).

**Table 1:** The novel-detected variant in the studied proband

**Gene**	**Variant**	**Zygosity**	**Associated phenotype**	**Mode of inheritance**	**ACMG classification**	**In silico predictions**	**PhyloP 100 score**	**PhastCons 100 score**
*PAX1* Exon 4/5	NM_006192.4:c.1212dup (p.Gly405Argfs*51)	Homozygous	OTFCS2	Autosomal recessive	Pathogenic	Pathogenic	5.324	1.000

## Discussion

Embryonic development requires the proper regulation of various transcription factors, whose dysfunctional activity could lead to serious congenital malformations (Wu et al. 2022). *PAX1* encodes a transcription factor protein that plays an essential role in different biological processes. It is especially expressed in embryogenesis during skeletal and pharyngeal pouch development, which gives rise to the thymus, tonsils, thyroid gland, and parathyroid glands (Thompson et al. 2021). Importantly, mouse point mutations in the paired-box domain of *PAX1* showed a dramatic decrease in protein DNA-binding affinity, leading to skeletal deformities. Further studies declared that PAX1 deficiency is correlated with moderate thymic hypoplasia in mice, which is more exacerbated when it is accompanied by Hoxa3 haploinsufficiency (Su et al. 2001).

**Fig. 3:**
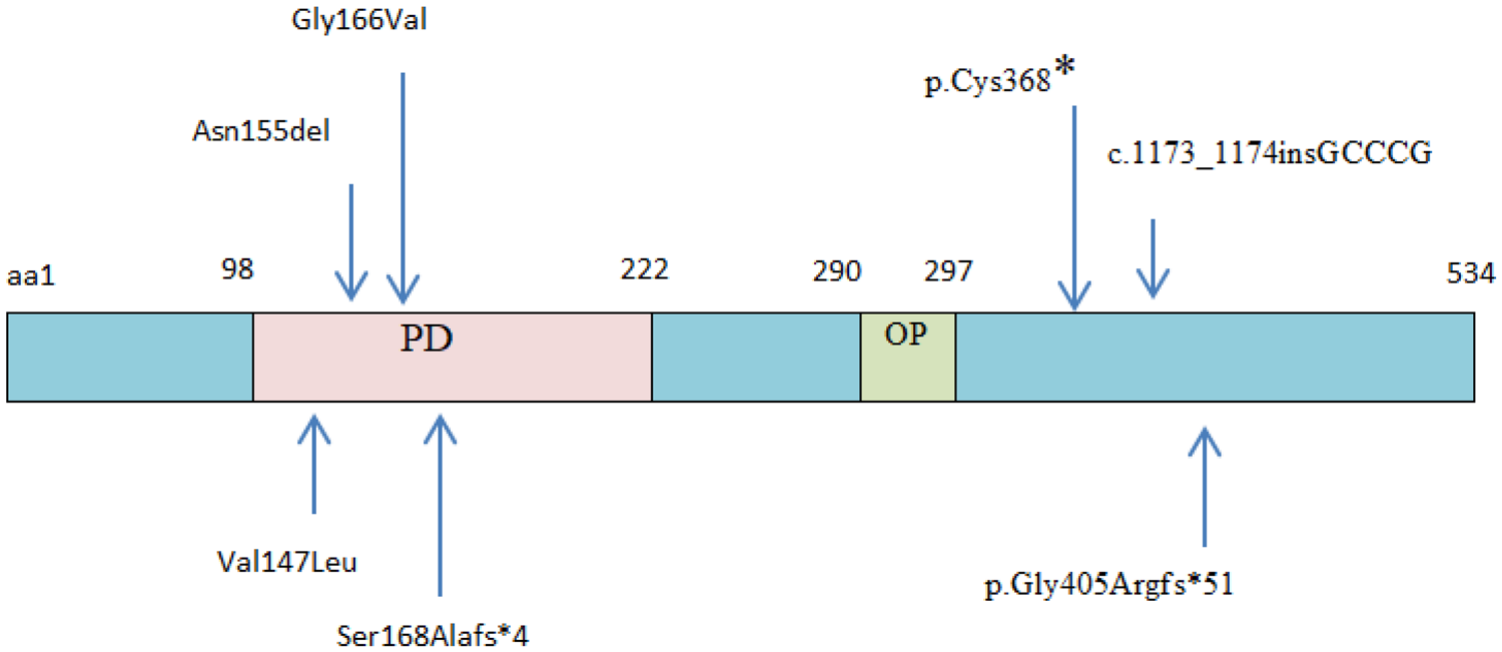
Schematic illustration of PAX1 protein showing the conserved paired-box domain (PD) as well as the octopeptide domain (OP). Positions of amino acids (aa) and locations of the identified *PAX1* variants related to OTFCS2 are indicated above

Human biallelic *PAX1* mutation is responsible for OTFCS2 disorder, while SCID is a variable aspect among the reported patients (Pohl et al. 2013; Yamazaki et al. 2020). To date, fourteen OTFCS2 cases from six unrelated families have been reported worldwide (Table 2) (Fig. 3). Four of these families were descendants from Middle Eastern countries.

**Table 2:** Clinical and molecular comparison in the currently studied family and others previously reported with OTFCS2 syndrome

	**Family (1) (Pohl et al. **2013)	**Family (2) (Paganini et al.** 2017**)**	**Family (3) (Patil et al.** 2018**)**	**Family (4) (Yamakazi et al.** 2020**)**	**Family (5) (Yamakazi et al.** 2020**)**	**Family (6) (Sherlaw-Sturrock et al. **2022)	**Family (7) (our study)**
*PAX1* mutation (NM_006192.4)	c.497G > T (p.Gly166Val)	c.1104C > A (p.Cys368*)	c.1173_1174 insGCCCG	c.463_465del p.Asn155del	c.439G > C (p.Val147Leu)	c.501del p.Ser168Alafs*4	c.1212dup (p.Gly405Argfs*51)
Number of cases	4	2	2	1	4	1	1
Gender	3 M, 1 F	1M, 1 F	1M/ 1F	M	1 M, 3 F	F	F
Ethnicity	Turkey	Morocco	India	German	Saudi Arabia	Iraq	Egypt
Intellectual disability	+ ve	ND	+ ve	ND	ND	− ve	+ ve
Developmental delay	+ ve	ND	+ ve	ND	ND	− ve	+ ve
Delayed speech	ND	ND	ND	ND	ND	ND	+ ve
Facial features							
Downslanting palpebral fissures	− ve	− ve	+ ve	ND	+ ve	− ve	+ ve
Microretrognathia	+ ve	+ ve	ND	+ ve	+ ve	− ve	− ve
Dysmorphic	+ ve	+ ve	+ ve	+ ve	+ ve	+ ve	+ ve
Hypertelorism	ND	+ ve	+ ve	ND	ND	+ ve	+ ve
Frontal bossing	ND	+ ve	ND	ND	+ ve	− ve	− ve
Facial asymmetry	ND	+ ve	− ve	ND	+ ve	− ve	− ve
Syndactyly	+ ve	ND	− ve	ND	ND	− ve	− ve
Depressed nasal bridge	ND	+ ve	ND	ND	+ ve	+ ve	+ ve
Long lashes	+ ve	− ve	+ ve	ND	ND	+ ve	+ ve
Ear							
Preauricular pits	+ ve	+ ve	− ve	ND	ND	+ ve	− ve
Low set ears	+ ve	+ ve	+ ve	ND	+ ve	ND	+ ve
Bilateral microtia	ND	+ ve	+ ve	+ ve	+ ve	+ ve	+ ve
Hearing loss	+ ve	ND	+ ve	ND	ND	+ ve	+ ve
Malformed pinna	+ ve	+ ve	+ ve	ND	+ ve	+ ve	+ ve
Skeletal							
Sloping shoulders	− ve	ND	− ve	ND	ND	ND	+ ve
Protruding shoulders	+ ve	ND	+ ve	ND	ND	ND	+ ve
Kyphosis	− ve	ND	+ ve	+ ve	ND	+ ve	− ve
Nail dystrophy of legs toes	ND	ND	ND	ND	ND	ND	+ ve
Winged scapulae	+ ve	ND	+ ve	ND	+ ve	ND	− ve
Eye							
Alacrima	+ ve	ND	ND	ND	ND	− ve	− ve
Immunodeficiency	ND	+ ve	− ve	+ ve	+ ve	+ ve	− ve
HBC transplantation (age)	ND	+ ve (3 months, 4 months)	ND	+ ve (12 months)	+ ve in 2 cases (12, 4 months)	+ ve (5 months)	− ve
Alive/death (age)	Alive (ND); however, the published images of patients showed late childhood and teenage life stages	Death at 9 months Death at 4 years and 7 months	Death at few months Alive at 2 years and 10 months	Alive at 6 years and 4 months	Alive at 6 years and 4 months Alive at 4 years and 4 months	Alive at 11 years and 3 months	Alive at 13 years

*ND* no data

In 4 out of the 6 previously reported families, OTFCS2 was associated with the T^−^ B^+^ NK^+^ SCID phenotype related to an underdeveloped or absent thymus. These cases had an Omenn syndrome-like phenotype, eosinophilia, and erythroderma as well as severe bacterial infections in their early life. They failed to achieve T-cell reconstitution after allogeneic hematopoietic stem cell (HPS) transplantation, leading to early death, postulating thymus transplantation as more appropriate management for OTFCS2 patients with SCID (Yamazaki et al. 2020).

Alternatively, patients of the other 2 families (*n* = 6) were reported to be free of SCID with no history of immunodeficiency (Pohl et al. 2013; Patil et al. 2018). Consistently, neither recurrent infections nor a history of SCID manifested in our proband (13 years old). Moreover, the patient revealed a normal complete blood picture, where both WBC and lymphocyte counts were within the normal ranges (5700 and 1208 per cubic millimeter, respectively). Normally, T cells comprise 70% of the circulating lymphocytes, so the decreased number of T cells in children with SCID usually leads to lymphopenia (Rivers and Gaspar 2015). Notably, the current patient has a reduced eosinophil count (46 per cubic millimeter), in contrast to eosinophilia reported in the T^−^ B^+^ NK^+^ SCID phenotype.

In the current study, whole exome sequencing revealed a novel homozygous variant (NM_006192.4:c.1212dup) creating a premature stop codon in the last exon. When translation terminates either in the last exon or near the last exon–exon junction in the penultimate exon, mRNA avoids nonsense-mediated decay (NMD), possibly due to the removal of the exon junction complex (EJC) from the last exon junction (Embree et al. 2022). Therefore, residual protein activity might be attained in the reported patient associated with proper immune functioning. Hypofunctional *PAX1* protein may be sufficient to stimulate the growth of the thymic epithelial lining from the third pharyngeal pouch. This might explain why some gene mutations might not be associated with congenital athymia. Therefore, we hypothesize that the activity of the mutant protein varies according to the variant type and location, which mediates whether SCID is progressed in OTFCS2 patients or not. This finding could be supported as the immune activity was similar among patients derived from the same family.

## Conclusion

*PAX1* mutations are generally associated with skeletal deformities that are highly comparable among all reported patients. However, the SCID is a variable aspect, where the hypofunctional *PAX1* protein might be adequate to drive thymus development and activity. Molecular studies on the effects of various *PAX1* variations on thymus tissue would provide useful insights into the disease genotype–phenotype correlation.

## Funding

This work has been funded by the NRC grant 12060187, Egypt.

## Data Availability

All study data are upon request and can be accessed by contacting the corresponding author.

## Declarations

Helsinki’s guiding principles were followed during the study. The study has been granted approval by the NRC Medical Research Ethics Committee (ID: 19261). After fully explaining the study to parents, their written agreement was acquired.
